# The diagnostic accuracy of pericardial and urinary lipoarabinomannan (LAM) assays in patients with suspected tuberculous pericarditis

**DOI:** 10.1038/srep32924

**Published:** 2016-09-16

**Authors:** Shaheen Pandie, Jonathan G. Peter, Zita S. Kerbelker, Richard Meldau, Grant Theron, Ureshnie Govender, Mpiko Ntsekhe, Keertan Dheda, Bongani M. Mayosi

**Affiliations:** 1 The Cardiac Clinic, Department of Medicine, Groote Schuur Hospital and University of Cape Town, Cape Town, South Africa; 2 TB Vaccine Group, Jenner Institute, University of Oxford, Oxford, UK; 3 Lung Infections and Immunity Unit and UCT Lung Institute, Division of Pulmonology, Department of Medicine, Groote Schuur Hospital, Cape Town, South Africa; 4 University of Cape Town, Cape Town, South Africa; 5 Institute of Infectious Diseases and Molecular Medicine, University of Cape Town, Cape Town, South Africa

## Abstract

We evaluated the diagnostic accuracy of urinary and pericardial fluid (PF) lipoarabinomannan (LAM) assays in tuberculous pericarditis (TBP). From October 2009 through September 2012, 151 patients with TBP were enrolled. *Mycobacterium tuberculosis* culture and/or pericardial histology were the reference standard for definite TBP. 49% (74/151), 33.1% (50/151) and 17.9% (27/151) of patients had definite-, probable-, and non-TB respectively; 69.5% (105/151) were HIV positive. LAM ELISA had the following sensitivity, specificity, positive likelihood ratio, negative likelihood ratio, positive predictive value and negative predictive values (95% confidence interval): urinary - 17.4% (9.1–30.7), 93.8% (71.7–98.9), 2.8 (0.1–63.3), 0.9 (0.8–0.9), 88.9% (56.5–98.0), and 28.3% (17.9–41.6); PF - 11.6% (6.0–21.3), 88% (70.0–95.8), 0.9 (0.08–12.0), 1.0 (0.9–1.1), 72.7% (43.4–90.1), and 26.6% (18.2–36.9). Sensitivity increased with a CD4 ≤ 100 cells/mm^3^ from 3.5% to 50% (p < 0.001) for urinary LAM ELISA; for urinary LAM strip test, grade 1 and 2 cut-points performed similarly, irrespective of HIV status or CD4 count. For PF LAM strip tests, switching cut-points from grade 1 to 2 significantly reduced test sensitivity (54.5% versus 19.7%; p < 0.001). Urinary and PF LAM assays have low sensitivity but high specificity for diagnosis of TBP. The sensitivity of urinary LAM is increased in HIV-infected patients with a CD4 ≤ 100 cells/mm^3^.

In sub-Saharan Africa, the syndemic of human immunodeficiency virus (HIV) and tuberculosis (TB) places immunosuppressed patients at greater risk of mortality and morbidity from extra-pulmonary forms of TB, including tuberculous pericarditis (TBP)^1^. The diagnosis of TBP remains problematic, as pericardial fluid may be paucibacillary, and culture and smear-based diagnostics perform sub-optimally^2^. The ddefinitive diagnosis of TBP is thus challenging and often delayed, making the evaluation of novel, rapid diagnostic tests essential^3^.

The *Mycobacterium tuberculosis (Mtb*) cell envelope is a source of distinctive glycoconjugates that are diagnostic and therapeutic targets^4^. The 17.5 kD glycolipid, lipoarabinomannan (LAM), found in the outer cell-wall of mycobacterial species has been investigated as a diagnostic antigen. LAM is released from metabolically active or degrading bacterial cells during TB infection^5^,^6^. The performance of urinary LAM in unselected outpatient TB suspects is poor, with moderate specificity and low sensitivity^7^,^8^. However, diagnostic performance of urinary LAM is improved in HIV infected patients with advanced immunosuppression (sensitivity of 21–62% in HIV-infected patients with suspected TB)^9^,^10^,^11^. Shah *et al.* found the overall sensitivity of the Clearview^®^ LAM ELISA amongst confirmed TB cases to be 59% in a nested cohort study of 499 South African in-patients with suspected TB (85% HIV-infected)^12^. The sensitivity improved to 71% (51–87%) in patients with a CD4+ T cell count of 50–100 cells/mm^3^, and 85% (73–93) in patients with CD4+ T cell count <50 cells/mm^3^; the urine LAM sensitivity in the smear negative TB patient group was 56%.

Although the majority of studies have evaluated the diagnostic utility of the LAM ELISA using urine in different clinical settings, the detection of LAM antigen has now been explored in several studies using various biological samples including serum, sputum, cerebrospinal fluid and pleural fluid^13^,^14^,^15^,^16^,^17^,^18^. To the best of our knowledge, no data exist on the utility of LAM for the diagnosis of pericardial tuberculosis.

Despite the sub-optimal performance of LAM as a diagnostic marker of TB, the ability to perform a rapid rule-in test for *Mtb* using an easily obtainable biological sample (such as urine) remains an attractive diagnostic option. More recent studies have also suggested that LAM may be used as a biomarker of prognosis, specifically in severely immunocompromised HIV infected patients^11^,^19^.

The aim of this study was to assess the diagnostic accuracy of the Determine^®^ TB Antigen lateral flow test and the LAM Clearview^®^ TB ELISA (not commercially available) in urine and pericardial fluid of suspected cases of TBP.

## Results

### Clinical characteristics

**Figure 1:**
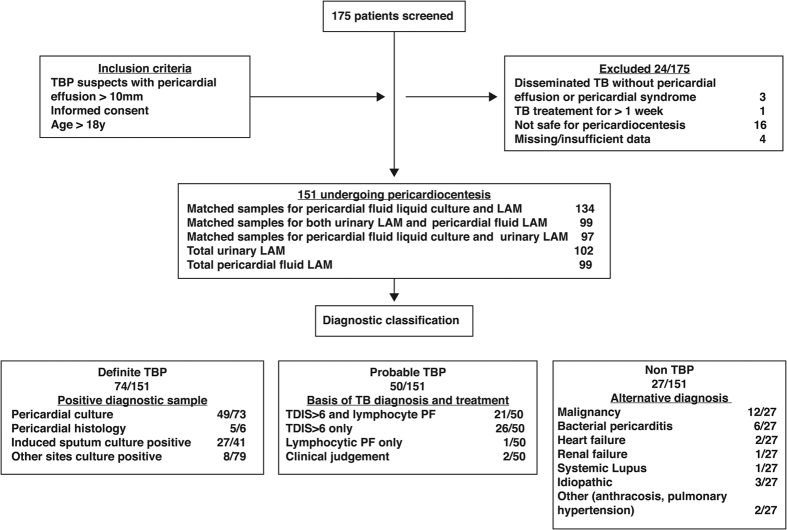
Screening, recruitment and diagnostic classification.

The study flow chart is illustrated in Fig. 1. 175 patients were screened for inclusion in the study. 24 patients were excluded on the basis of having a pericardial effusion that was not amenable to safe pericardiocentesis (n = 16), missing information (n = 4), absence of pericardial effusion (n = 3) and prolonged anti-TB therapy (n = 1). Of the remaining 151 included patients, 49% (74/151), 33.1% (50/151) and 17.9% (27/151) were classified as definite-, probable-, and non-TB, respectively.

Table 1A,B highlight the demographic, clinical, echocardiographic and biochemical characteristics of patients with suspected TBP stratified by final diagnostic classification. 69.5% (105/151) of patients were HIV positive (median (interquartile range (IQR)) CD4+ T cell count 139 (81 to 249) cells/mm^3^, 24.5% were HIV negative (37/151), and 6% (9/151) of unknown HIV status.

### Diagnostic accuracy measures

Table 2 compares the diagnostic accuracy measures for the biomarker diagnosis of TBP, stratified by HIV-status and CD4+ T cell count. Definite-TB was used to calculate sensitivity and non-TB was used for specificity. Detailed results of the diagnostic accuracy of PF unstimulated interferon γ (uIFNγ) levels as measured by the InterGam Ultrasensitive Rapid Immuno-suspension Assay (IRISA; Antrum Biotech, Cape Town, South Africa; www.antrumbiotech.com) are published elsewhere^3^. In summary, at a cut-point of ≥44 pg/ml, PF uIFNγ had a sensitivity, specificity, positive likelihood ratio (LR+), negative likelihood ratio (LR−), positive predictive value (PPV) and negative predictive value (NPV) (95% confidence interval (CI)) of 95.7% (88.1–98.5), 96.3% (81.7–99.3), 25.8 (3.7–184), 0.045 (0.023–0.09), 91.7% (88.1–94.3) and 98.1% (96.8–98.9), respectively. There was no significant difference in the diagnostic accuracy of PF uIFNγ by HIV status. PF ADA levels performed similarly but with a significantly lower specificity (96.3% versus 84%; p = 0.03), LR+ (25.8 versus 6.0; p = 0.03), and PPV (91.7% versus 71.9%; p = 0.03). PF ADA had a higher specificity in HIV negative versus positive patients (100% (12/12) versus 40% (2/5); p = 0.003).

### LAM ELISA assays

Urinary LAM ELISA had a sensitivity, specificity, LR+, LR−, PPV and NPV (95% CI) of 17.4% (9.1–30.7), 93.8% (71.7–98.9), 2.8 (0.1–63.3), 0.9 (0.8–0.9), 88.9% (56.5–98.0), and 28.3% (17.9–41.6) respectively. The test sensitivity increased significantly in patients with a CD4+ T cell count <100 cells/cm^3^ (3.5% versus 50%; p = 0.0004).

PF LAM ELISA had a sensitivity, specificity, LR+, LR−, PPV and NPV (95% CI) of 11.6% (6.0–21.3), 88% (70.0–95.8), 0.9 (0.08–12.0), 1.0 (0.9–1.1), 72.7% (43.4–90.1), and 26.6% (18.2–36.9) respectively. While having a high specificity in both HIV reactive and non-reactive patients, the sensitivity was equally poor in both groups. The test sensitivity increased significantly in patients with a CD4+ T cell count <100 cells/cm^3^ (27.2% versus 5.1%; p = 0.01).

There was no difference in the diagnostic utility of urinary versus PF LAM ELISA, irrespective of HIV status or level of immunosuppression. The test sensitivity of the urinary and pericardial LAM (ELISA and strip test formats) did not change when the definite-TB group was restricted to pericardial culture positive patients only (data not shown).

### LAM strip tests

**Figure 2:**
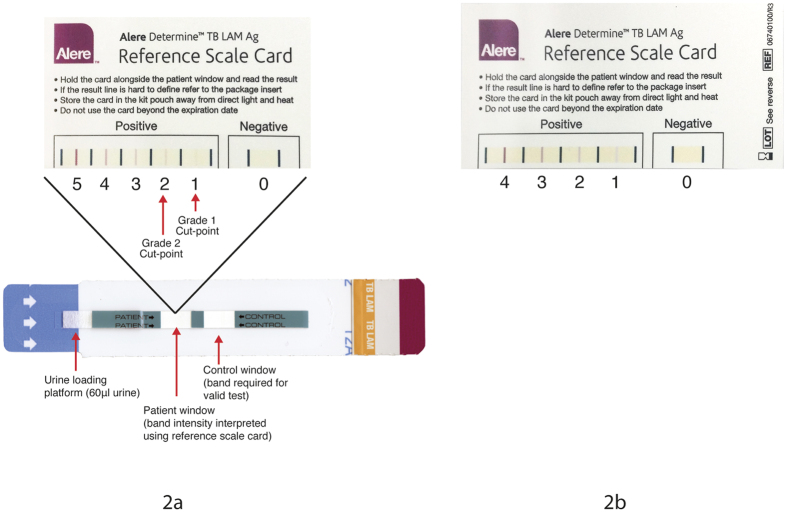
(**a**) Pre-January 2014 LAM strip test manufacturer’s reference card illustrating visual intensity grades 0–5; (**b**) January 2014 new LAM strip test manufacturer’s reference card illustrating visual intensity grades 0–4. In this reference card the first positive band corresponds to the grade-2 intensity band in the old, pre-January 2014 reference card. Permission granted by Alere to publish this figure.

The diagnostic accuracy of point-of-care LAM strip tests was evaluated for both the grade 1 and 2 cut-points (Fig. 2). For urinary LAM strip test, the diagnostic performance was similar for grade 1 and 2 cut-points, irrespective of HIV status or CD4+ T cell count. However, urinary LAM strip test (cut-point 2) sensitivity increased significantly in patients with a CD4+ T cell count <100 cells/cm^3^ (50% versus 18.5%; p = 0.04).

For PF LAM strip tests, switching cut-points from grade 1 to 2 significantly decreased the test sensitivity in all patients (54.5% versus 19.7%; p < 0.001), and the HIV positive group (58.5% versus 20.8%; p < 0.001).

The test sensitivity of the urinary and pericardial LAM (ELISA and strip test formats) did not change when the definite-TB group was restricted to pericardial culture positive patients only (data not shown).

The addition of the probable-TB group to the definite-TB patients for the calculation of sensitivity did not alter the conclusions for either urinary or pericardial LAM (ELISA and strip test formats) (Table S1 in the supplementary appendix).

### Comparative diagnostic accuracy

PF ADA and PF uIFNγ sensitivity was consistently superior to all variations of LAM testing: ADA and uIFNγ 95.7%; urinary LAM ELISA 17.4%; urinary strip test (grade 1 and 2) 26.7%; PF LAM ELISA 11.6%; PF LAM strip test (grade 1) 58.5%; PF LAM strip test (grade 2) 19.7% (p < 0.001 for all comparisons of sensitivity between ADA and uIFNγ versus LAM tests). There was no significant difference between the specificities of the biomarkers evaluated.

When using the grade 1 cut-point, the PF LAM strip test outperformed the PF LAM ELISA with a significantly better sensitivity overall (54.5% versus 11.6%; p < 0.001), as well as in the HIV positive (58.5% versus 27.3%; p < 0.001) and HIV negative (38.5% versus 0.0%; p = 0.01) groups. The sensitivity of the grade 1 cut-point was also significantly better than the grade 2 cut-point (All patients: 54.5% versus 19.7%, p < 0.001; HIV positive: 58.5% versus 20.8%, p < 0.001; CD4+ T cell count <100 cells/cm^3^: 68.2% versus 27.3%, p = 0.006).

## Discussion

The key findings are that: (i) both urine LAM ELISA and strip testing had poor sensitivity, but were able to diagnose TBP in half of patients with CD4+ T cell count ≤100 cell/cm^3^; and (ii) PF LAM testing had poor sensitivity, irrespective of HIV or CD4+ T cell count, and offers little utility over existing PF biomarkers. Thus, as in other forms of extra-pulmonary or disseminated TB in severely immunocompromised patients, urinary LAM may serve as a simple non-invasive rule-in diagnostic tool where TB is suspected but difficult to confirm^20^.

Urinary LAM testing is an attractive diagnostic tool – simple to perform on an easily obtainable biological sample. It has been shown to be a useful diagnostic tool in a select population of HIV-infected patients with advanced immunosuppression. Mutetwa *et al.* showed that LAM ELISA sensitivity was significantly higher in patients with HIV-related TB, while Balcha *et al.* and Peter *et al.* confirmed that the test accuracy improved with decreasing CD4 counts^7^,^9^,^10^.

70% of our cohort was HIV infected, with advanced immunosuppression – clinically stage 4 HIV with extra-pulmonary TB (suspected or confirmed TBP), and a median CD4+ T cell count of less than 150 cells/cm^3^. The diagnostic performance of both urinary LAM ELISA and urinary strip tests were comparable to previously published studies, showing excellent potential for rule-in, but poor sensitivity. As predicted, the test sensitivity improved as CD4+ T cell counts decreased, with the best utility being in the HIV positive group with CD4 counts less than 100 cells/cm^3^. The major advantage of LAM diagnostics would be to rule in TB in cases that are clinically challenging, or specific biological samples (such as pericardial fluid) are difficult to obtain. Despite improvement in LAM sensitivity in HIV-positive patients with low CD4+ T cell count, clinical scoring systems (such as the Tygerberg TB Pericarditis Diagnostic Index) still out-performed urine-based LAM testing for the suspected diagnosis of TBP (Table 3)^3^. Combining urine LAM with either of the clinical predictors did not improve the diagnostic accuracy (Table S1 in supplementary appendix). In high-TB-HIV prevalent settings, a test that can confidently rule out TBP is more appropriate, especially for clinically challenging cases.

We evaluated the novel application of PF to both Clearview^®^ TB ELISA assays and the Determine^®^ TB lateral flow point-of-care strip tests. Again, both the LAM ELISA and strip test had good specificity, but low sensitivity. PF LAM ELISA was inferior to current available PF biomarkers such as ADA and uIFNγ (InterGam). This is likely related to the pauci-bacillary nature of the majority of cases of TBP, and the fact that TB pericardial effusions are the result of the predominant T-helper 1 immune inflammatory response, creating a lymphocytic exudate with a cytokine and enzyme milieu rich in IFNγ, tumour necrosis factor (TNF) alpha, interleukin-15 (IL) and ADA^21^,^22^,^23^.

The ability to perform a point-of-care TBP diagnostic test using pericardial samples is very appealing. Our group has published data showing that the rapid bacteriological diagnosis of TBP was possible in two thirds of TBP suspects using the new WHO-endorsed Xpert MTB/RIF when applied to PF, and that uIFNγ levels measured by the InterGam Ultrasensitive Rapid Immuno-suspension Assay was most accurate for biochemical diagnosis^3^. However, with uIFNγ point-of-care test still under production (Antrum Biotech, Cape Town, South Africa; www.antrumbiotech.com), and in areas where Xpert MTB/RIF, uIFNγ, or ADA assays are not available, point-of-care LAM strip testing on PF may be a useful diagnostic tool.

LAM strip tests were evaluated using the pre-January 2014 manufacturer’s reference card (Supplementary Figure S1A). When applied to PF, there was a significance difference between LAM sensitivity, with no change in the specificity, when using the grade 1versus the grade 2 cut-point. This was consistent with previously published data evaluating grade 1 versus grade 2 cut-points for urinary LAM strip tests^10^. Since January 2014, the manufacturers have revised the reference card, changing from 5 grades to 4 (Supplementary Figure S1B). The updated reference card eliminates the grade 1 cut-point, making the first positive band correspond to the grade 2 cut-point – essentially meaning that the currently available LAM strip test would perform at the lower sensitivity when applied to PF.

Our study had a number of important limitations. Whilst this is largest study that has comprehensively evaluated serial diagnostic strategies and tools in the same prospective cohort, and the first study to evaluate LAM as a diagnostic tool in suspected TBP patients, the sample size was limited in the non-TB group. The small number of non-TB patients reflects the high burden of TB and HIV in the South African environment^24^. Furthermore, quantitative analysis of urinary LAM has been shown to have prognostic value in patients with HIV-associated TB^19^. Our sample size and follow-up period was insufficient to explore the possibility of LAM being a prognostic marker in TBP.

## Conclusion

In conclusion, urinary and PF LAM ELISA tests are inferior to the currently available bacteriological, serological and clinical diagnostic tools for TBP because of low sensitivity. High TB-HIV prevalence and frequent empiric TB treatment strategies for TBP, increase the need for a rapidly available point-of-care rule-out tests, further limiting the potential of TBP LAM based diagnostics.

## Methods

### Study population

Between October 2009 and September 2012, consecutive patients with suspected TBP referred to Groote Schuur Hospital in Cape Town for enrolment in the Investigation of Management of Pericarditis in Africa (IMPI) registry and trial were screened for inclusion in this diagnostic study^25^,^26^. Inclusion criteria were the presence of a pericardial effusion with an echo-free space of greater than 10 mm around the heart in diastole, age 18 years or older, and provision of informed consent. Patients were excluded if they: (i) received more than 1 week of anti-TB therapy prior to pericardiocentesis; (ii) were pregnant; or (iii) were unable to provide informed consent. The methods of the study were carried out in accordance with the approved guidelines. Written informed consent was obtained from each patient prior to enrolment, and the study protocol conforms to the ethical guidelines of the 2008 Declaration of Helsinki as reflected in *a priori* approval by the human research ethics committee of the University of Cape Town (HRECREF402/2005). The design and methods of this study have been published elsewhere^3^.

### Diagnostic sample collection and handling

A minimum of 60 ml of pericardial fluid (PF) was collected by percutaneous pericardiocentesis for diagnostic testing, by routine (i.e., adenosine deaminase (ADA); lactate dehydrogenase levels (LDH); concentrated fluorescence smear microscopy and mycobacteria growth indicator tube (MGIT) liquid culture (MGIT 960, BD Diagnostic, Hunt Valley, MD, USA) and novel methods (i.e., LAM assays and lateral flow point-of-care strip test; Xpert MTB/RIF assay; and unstimulated interferon-γ (uIFNγ levels)). Routine biomarker and pericardial fluid smear and culture were performed by the accredited National Health Laboratory Service, while the Lung Infection and Immunity research laboratory performed LAM assays and strip tests, Xpert MTB/RIF and uIFNγ testing.

Details of the performance and results Xpert MTB/RIF and uIFNγ levels are published elsewhere^3^. Patients were requested to submit two urine specimens. Both unprocessed and centrifuged urine and pericardial fluid samples, respectively, were stored at −20 °C for batch LAM testing within 12 months of collection. Clearview^®^ TB LAM ELISA and Determine^®^ TB strip test (Alere, USA) were both performed according to manufacturers’ instructions on 100 μl of thawed unprocessed urine, and processed PF. ELISA plates were read immediately at 450 nm on an ELISA plate reader. Positive and negative controls were run in duplicate. Average optical density (OD) readings from duplicate samples normalized to the OD of the negative control were generated. LAM strip tests were loaded with 60 μl of urine and graded by two independent readers after 25 minutes using the pre-January 2014 manufacturer’s reference card. A 3^rd^ reader provided consensus if necessary. Readers were blinded to results of reference tests.

### Diagnostic classification for analysis

All patients that were included had a clinical diagnosis of a pericarditis or a pericardial syndrome, with echocardiographic confirmation of a pericardial effusion. Pericardial syndrome was defined as:

- Clinical features of pericarditis including chest pain and shortness of breath;
- Clinical features of heart failure in the setting of a confirmed pericardial effusion;
- Compatible ECG and or echocardiographic findings of a pericarditis and/or pericardial effusion.

Patients were categorised into the following diagnostic groups based on a combination of pericardial and non-pericardial sample culture results, histopathology of pericardial biopsy samples, basic pericardial fluid characteristics, and the commencement of TB treatment:

- Definite-TB: At least one *Mtb* sample positive by liquid culture (either pericardial or non-pericardial) and/or granulomatous inflammation on pericardial tissue histology (i.e., composite reference standard). Non-pericardial samples included sputum, lymph node biopsy, pleural fluid, blood, cerebrospinal fluid and blood.
- Probable-TB: Clinical (Tygerberg TB Pericarditis Diagnostic Index Score (TDIS) of ≥6) and/or biochemical diagnosis characterised by elevated ADA and lymphocytic predominance; not meeting the criterion for definite-TB, but given TB treatment. The TDIS is a weighted score comprising clinical variables (weight loss, night sweats and fever) and blood biochemistry (serum globulin >40 g/l and leukocyte count <10 × 10^9^/l)^27^.
- Non-TB: No microbiological evidence of *M. tb* and an alternative diagnosis found.

### Statistical analysis

A univariate analysis was used to determined basic clinical predictors of definite-TB pericarditis. A set of multivariate clinical predictors was generated using logistic regression modeling. Multiple imputation by chained equations was used to impute missing data prior to model building^28^. Using receiver operator characteristics (ROC) analysis the continuous variable, age, was dichotomised to maximise discriminatory utility to generate a binary variable (age >40 years). Rounded ß-coefficients from the reduced model of significant variables were used to generate scores to quantitate relevant clinical predictors included in the final model. ROC analysis was performed and three cut-points were selected for rule-in, Youden’s index (the optimal mathematical balance between sensitivity and specificity) and rule-out value^29^. Diagnostic accuracy, including 95% confidence intervals, for each cut-point was assessed using sensitivity, specificity, positive and negative predictive values (PPV, NPV) and positive and negative likelihood ratio (LR+, LR−). STATA IC, version 10 (Stata Corp, Texas, USA) was used for all statistical analyses. Sensitivity, specificity, LR+, LR−, PPV) and NPV for all diagnostic tests are presented with 95% confidence intervals (CI).

Demographic, clinical and microbiological characteristics of different diagnostic groups were compared using χ^2^ and Wilcoxon rank-sum tests as appropriate. Diagnostic sensitivity and specificity of individual or combined tests were compared using the χ^2^ and Fisher’s exact tests as appropriate. The Spearman correlation coefficient (R_s_) was used to evaluate the association between LAM concentration and reported outcomes, for both urinary LAM and pericardial LAM. All statistical tests were two sided at α = 0.05 and analyses were performed using STATA IC, version 10 (Stata Corp, College Station, TX, USA). The STARD criteria were used for analysis and reporting of this study^30^.

## Additional Information

**How to cite this article**: Pandie, S. *et al.* The diagnostic accuracy of pericardial and urinary lipoarabinomannan (LAM) assays in patients with suspected tuberculous pericarditis. *Sci. Rep.* **6**, 32924; doi: 10.1038/srep32924 (2016).

## Supplementary Material

Supplementary Appendix

## Figures and Tables

**Table 1:** Baseline demographic and clinical (A), echocardiographic and biochemical (B) characteristics of patients referred with suspected TB pericarditis.

	All	Definite-TB	Probable-TB	Non-TB
	N = 151	n = 74	n = 50	n = 27
**A.**				
**Demographics**				
Age (median, IQR)	34 (29–42)	33 (27–38)*	33 (28–37)*	52 (34–60) *p < 0.001
Male (n, %)	93 (62)	48 (65)	32 (64)	13 (48)
HIV positive (n, %)¥	105 (74)	59 (80)*	41 (82)*	5 (28) *p < 0.001
CD4 count (median, IQR)¥	139 (81–249)	131 (70–206)*	153 (81–271)*	301 (229–424) *p = 0.04
ARV therapy (n/N, %)	18/98 (18)	10/54 (19)	7/39 (18)	1/5 (20)
**Presenting clinical features**				
NYHA Class I–II (n/N, %)	77/134 (58)	39/66 (59)*	35/47 (75)*	3/21 (14) *p < 0.001
NYHA Class III–IV (n/N, %)	57/134 (41)	27/66 (41)*	12/47 (25)*	18/21 (86) *p < 0.001
Systolic Blood Pressure (mean, SD) (n = 145)	113 (17)	113 (17)	112 (16)	115 (22)
Diastolic Blood Pressure (mean, SD) (n = 145)	72 (14)	72 (15)	72 (13)	73 (14)
Heart rate (mean, SD) (n=146)	111 (20)	114 (22)	108 (16)	111 (21)
**Serum biochemical data**				
Haemoglobin g/dl (mean, SD)	9.6 (2.1)	9.4 (2.1)*	9.3 (1.7)*	10.9 (2.3) *p = 0.004
Creatinine, μmol/l (median, IQR)	73 (59–90)	72 (58–86)	77 (61–92)	72 (61–81)
Total WCC × 10^9^/l (median, IQR)	6.5 (4.8–9.3)	6.5 (4.4–8.2)*	5.8 (4.8–7.2)*	10.5 (7.1–15.2) *p < 0.001
**B.**				
**Echocardiographic features**				
Size of effusion (mm)(mean, SD) (n = 126)	36 (14)	36 (14)*	38 (13)*	29 (14) *p < 0.001
Tamponade (n, %) (n = 141)	94 (67)	50 (72)	32 (64)	12 (55)
**Routine pericardial fluid analyses**				
ADA IU/l (median, IQR) (n = 142)	51 (34–75)	59 (45–86)*	51 (34–77)*	17 (11–27)* p < 0.001
Total protein g/l (median, IQR) (n = 148)	60 (52–68)	59 (53–68)	63 (56–68)	56 (48–62)
Lactate Dehydrogenase (median, IQR) (n = 135)	1419 (867–2305)	1553 (999–2800)*	1093 (725–1613)*	884 (442–2305) *p = 0.02
Total PF WCC × 10^9^/l (Median, IQR) (n = 136)	2.1 (1.1–3.3)	2.0 (1.2–3.0)	2.2 (1.2–2.9)	3.0 (0.7–8.9)
Lymphocyte predominance (n/N, %)§	52/107(49)	29/56 (52)*	22/34 (65)*	1/17 (6) *p < 0.001

^¥^9 patients refused testing or had unknown HIV status, and 4 HIV-infected patients had no CD4 cell count data.

^*^P values indicate significant differences between patient groups.

^§^Multiple invalid results.

IQR, Inter-quartile range; SD, Standard deviation; ARV, Anti-retroviral therapy; NYHA, New York Heart Association; ADA, Adenosine deaminase; WCC, White cell count; PF, pericardial fluid.

**Table 2:** Diagnostic accuracy measures of urinary and pericardial fluid LAM Clearview^®^ TB ELISA and the Determine^®^ TB lateral flow point-of-care strip test (definite-TB for sensitivity and non-TB for specificity calculations).

Diagnostic Test	Patient group	Sensitivity (95% CI) (n/N)	Specificity (95% CI) (n/N)	Positive Likelihood ratio, LR+ (95% CI)	Negative Likelihood ratio, LR− (95% CI)	Positive predictive value, PPV (95% CI)	Negative predictive value, NPV (95%CI)
Urine LAM ELISA	All patients	17.4%* (9.1–30.7) 8/46	93.8% (71.7–98.9) 15/16	2.8 (0.1–63.3)	0.9 (0.8–0.9)	88.9% (56.5–98.0)	28.3% (17.9–41.6)
	HIV positive	21.6%* (11.4–37.2) 8/37	100% (34.2–100) 2/2	undefined	0.8 (0.7–0.8)	100% (67.6–100)	6.5% (1.8–20.7)
	CD4 <100 cells/mm^3^	50%* (25.4–74.6) 6/12	Undefined 0/0	undefined	undefined	100% (61.0–100)	0.0% (0.0–39.0)
	CD4 >100 cells/mm^3^	3.5%* (0.6–17.1) 1/28	100% (56.5–100) 5/5	undefined	1.0 (0.8–1.0)	100% (20.6–100)	15.6% (6.9–31.8)
	HIV negative	0.0%* (0.0–29.9) 0/9	90% (59.6–98.2) 9/10	0.0	1.1 (undefined)	0.0% (0.0–79.6)	50% (29.0–71.0)
Urine LAM strip test (grade 1 cut-point)	All patients	26.7%* (15.9–41.0) 12/45	91.7% (64.6–98.5) 11/12	3.2 (0.3–35.6)	0.8 (0.7–0.8)	92.3% (66.7–98.6)	25% (14.6–39.4)
	HIV positive	33.3%* (20.2–49.6) 12/36	100% (34.4–100) 2/2	undefined	0.7 (0.6–0.7)	100% (75.6–100)	7.6% (2.1–24.1)
	CD4 <100 cells/mm^3^	50%* (25.4–74.6) 6/12	Undefined 0/0	undefined	undefined	100% (61.0–100)	(0.0–39.0)
	CD4 >100 cells/mm^3^	18.5%* (8.1–36.7) 5/27	100% (51.0–100) 4/4	undefined	0.8 (0.7–0.9)	100% (56.6–100)	15.3% (6.2–33.6)
	HIV negative	0.0%* (0.0–29.9) 0/9	85.7% (48.7–97.4) 6/7	0.0 (undefined)	1.2 (undefined)	0.0% (0.0–79.4)	40% (19.8–64.3)
Urine LAM strip test (grade 2 cut-point)	All patients	26.7%* (15.9–41.0) 12/45	90.9% (62.2–98.4) 10/11	2.9 (0.3–32.6)	0.8 (0.7–0.9)	92.3% (66.7–98.6)	23.3% (13.2–37.7)
	HIV positive	33.3%* (20.2–49.6) 12/36	100% (34.4–100) 2/2	undefined	0.7 (0.6–0.7)	100% (75.6–100)	7.6% (2.1–24.1)
	CD4 <100 cells/mm^3^	50%* (25.4–74.6) 6/12	Undefined 0/0	undefined	undefined	100% (61.0–100)	0.0% (0.0–39.0)
	CD4 >100 cells/mm^3^	18.5%* (8.1–36.7) 5/27	100% (51.0–100) 4/4	undefined	0.8 (0.7–0.9)	100% (56.6–100)	15.3% (6.2–33.6)
	HIV negative	0.0%* (0.0–29.9) 0/9	85.7% (48.7–97.4) 6/7	0.0 (undefined)	1.2 (undefined)	0.0% (0.0–79.4)	40% (19.8–64.3)
PF LAM ELISA	All patients	11.6%*^¥^ (6.0–21.3) 8/69	88% (70.0–95.8) 22/25	0.9 (0.08–12.0)	1.0 (0.9–1.1)	72.7% (43.4–90.1)	26.6% (18.2–36.9)
	HIV positive	14.5%*^¥^ (7.6–26.2) 8/55	100% (51.0–100) 4/4	undefined	0.9 (0.8–0.9)	100% (67.6–100)	7.8% (3.1–18.5)
	CD4 <100 cells/mm^3^	27.3%*^¥^ (13.2–48.2) 6/22	Undefined (0/0)	undefined	undefined	100% (61.0–100)	0.0% (0.0–19.4)
	CD4 >100 cells/mm^3^	5.1%*^¥^ (1.4–16.9) 2/39	100% (67.6–100) 8/8	undefined	0.9 (0.9–1.0)	100% (34.3–100)	17.8% (9.3–31.3)
	HIV negative	0.0%*^¥^ (0.0–21.5) 0/14	100% (75.8–100) 12/12	undefined	1 (undefined)	undefined	46.2% (28.8–64.6)
PF LAM strip test (grade 1 cut-point)	All patients	54.5%*^¥§^ (42.6–65.9) 36/66	68% (48.4–82.8) 17/25	1.7 (1.2–2.3)	0.7 (0.6–0.8)	81.8% (68.0–90.5)	36.2% (24.0–50.5)
	HIV positive	58.5%*^¥§^ (45.1–70.7) 31/53	75% (30.1–95.4) 3/4	2.4 (0.3–17.4)	0.6 (0.4–0.8)	96.8% (84.3–99.5)	12% (4.2–30.0)
	CD4 <100 cells/mm^3^	68.2%*^¥§^ (47.3–83.6) 15/22	Undefined 0/0	undefined	undefined	100% (79.6–100)	0.0% (0.0–35.4)
	CD4 >100 cells/mm^3^	50%*^¥§^ (34.4–65.5) 18/36	75% (40.9–92.9) 6/8	2 (0.7–5.9)	0.7 (0.5–0.8)	90% (69.9–97.2)	25% (12.0–44.9)
	HIV negative	38.5%*^¥§^ (17.7–64.5) 5/13	50% (21.5–78.5) 4/8	0.8 (0.3–2.4)	1.2 (0.6–2.6)	55.5% (26.7–81.1)	33.3% (13.8–61.0)
PF LAM strip test (grade 2 cut-point)	All patients	19.7%*^§^ (11.9–30.8) 13/66	84% (65.4–93.6) 21/25	1.2 (0.4–3.7)	0.9 (0.9–1.0)	76.5% (52.7–90.5)	28.4% (19.4–39.5)
	HIV positive	20.8%*^§^ (12.0–33.5) 11/53	100% (51.0–100) 4/4	undefined	0.8 (0.7–0.8)	100% (74.1–100)	8.7% (3.4–20.3)
	CD4 <100 cells/mm^3^	27.3%*^§^ (13.1–48.1) 6/22	Undefined 0/0	undefined	undefined	100% (61.0–100)	Undefined
	CD4 >100 cells/mm^3^	19.4%*^§^ (9.8–35.0) 7/36	87.5% (52.9–97.8) 7/8	1.6 (0.06–35.2)	0.9 (0.8–1.0)	87.5% (52.9–97.8)	19.4% (9.8–35.0)
	HIV negative	15.4%* (4.3–42.3) 2/13	83.3% (55.2–95.3) 10/12	0.9 (0.0–539.1)	1.0 (0.8–1.2)	50% (15.0–85.0)	47.6% (28.3–67.6)
uIFNγ (Intergam) (Youden’s, rule-in and rule-out cut-points: ≥44 pg/ml)	All patients	95.7%* (88.1–98.5) 67/70	96.3% (81.7–99.3) 26/27	25.8 (3.6–184)	0.045 (0.023–0.09)	91.7% (88.1–94.3)	98.1% (96.8–98.9)
	HIV positive	98.2%* (90.6–99.7) 55/56	80% (37.6–96.4) 4/5	4.9 (0.7–34.9)	0.02 (0.003–0.179)	98.2% (90.5–99.7)	80% (37.6–96.4)
	HIV negative	100%* (78.5–100) 14/14	100% (77.2–100) 13/13	undefined	undefined	100% (78.5–100)	100% (77.2–100)
ADA (Cut-point in current clinical use: ≥35 IU/ml)	All patients	95.7%* (88.1–98.5) 67/70	84% (65.4–93.6) 21/25	6.0 (3.7–9.8)	0.051 (0.026–0.10)	71.9% (67.3–76.1)	97.9% (96.4–98.7)
	HIV positive	96.4%* (87.7–99.0) 53/55	40%^¶^ (11.8–76.9) 2/5	1.6 (0.8–3.1)	0.09 (0.007–1.05)	94.6% (85.4–98.2)	50% (15.0–85.0)
	HIV negative	93.3%* (70.2–98.8) 14/15	100%^¶^ (75.8–100) 12/12	undefined	0.067 (0.009–0.473)	100% (78.5–100)	92.3% (66.7–98.6)

^*^uIFNγ and ADA sensitivity was significantly better than urinary and PF LAM ELISA and strip tests, for all patient categories (p < 0.001).

^¶^ADA specificity was significantly higher in HIV negative patients (p = 0.003).

^¥^PF LAM strip test grade 1 sensitivity was significantly higher than PF LAM ELISA (All patients p < 0.001; HIV positive patients p < 0.001; CD4 <100 cells/mm^3^ p = 0.006; CD4 >100 cells/mm^3^ p < 0.001; HIV negative patients p = 0.01).

^§^PF LAM strip test grade 1 sensitivity was significantly better than PF LAM strip test grade 2 (All patients p < 0.001; HIV positive patients p < 0.001; CD4 <100 cells/mm^3^ p = 0.006; CD4 >100 cells/mm^3^ p = 0.006).

**Table 3:** Comparison of urinary LAM ELISA and strip tests with clinical diagnostics for diagnosing TBP (definite-TB for sensitivity and non-TB for specificity calculations).

Diagnostic Test	Sensitivity (95% CI) (n/N)	Specificity (95% CI% CI) (n/N)	Positive Likelihood ratio, LR+ (95% CI)	Negative Likelihood ratio, LR− (95% CI)	Positive predictive value, PPV (95% CI)	Negative predictive value, NPV (95% CI)
Urine LAM ELISA	17.4%* (9.1–30.7) 8/46	93.8% (71.7–98.9) 15/16	2.8 (0.1–63.3)	0.9 (0.8–0.9)	88.9% (56.5–98.0)	28.3% (17.9–41.6)
Urine LAM strip test (grade 2 cut-point)	26.7%* (15.9–41.0) 12/45	90.9% (62.2–98.4) 10/11	2.9 (0.3–32.6)	0.8 (0.7–0.9)	92.3% (66.7–98.6)	23.3% (13.2–37.7)
Tygerberg score ≥6	85.3%* (75.9–81) 58/68	77.3% (56.6–89.9) 17/22	3.75 (2.52–5.59)	0.19 (0.15–0.24)	61.7% (56.9–66.2)	92.5% (90.0–94.3)
IMPI Clinical predictors (rule-in cut-point >6.1)	60.8%* (49.4–71.1) 45/74	96.3% (81.7–99.3) 26/27	16.4 (2.25–119.9)	0.41 (0.38–0.44)	87.6% (82.4–91.4)	85.1% (82.5–87.5)
IMPI Clinical predictors (Youden’s and rule-out cut-point >3.5)	91.9%* (83.4–96.2) 68/74	81.5% (63.3–91.8) 22/27	4.96 (3.34–7.36)	0.1 (0.07–0.14)	68.0% (63.3–72.4)	95.9% (94.0–97.2)

^*^Tygerberg score ≥6 and IMPI Clinical predictors (rule-in cut-point >6.1 and rule-out cut-point >3.5) had a significantly greater sensitivity (p < 0.001).
