# Supplementary material for: The diagnostic accuracy of pericardial and urinary lipoarabinomannan (LAM) assays in patients with suspected tuberculous pericarditis
Source: Sci Rep. 2016 Sep 16;6:32924. doi: 10.1038/srep32924 (PMC5025647; doi:10.1038/srep32924)
Supplement: Supplementary Appendix [file srep32924-s1.doc]

# Supplementary Appendix:

# The Diagnostic utility of pericardial and urinary Lipoarabinomannan (LAM) assays in patients with suspected tuberculous pericarditis

Shaheen Pandie^1^*; Jonathan G. Peter^2,3^*; Richard Meldau^3^ ; Zita S. Kerbelker^1^; Grant Theron^3^; Ureshnie Govender^3^; Mpiko Ntsekhe^1^; Keertan Dheda^3,4^*; Bongani M Mayosi^1^*

*These authors contributed equally to this work.

^1^The Cardiac Clinic, Department of Medicine, Groote Schuur Hospital and University of Cape Town, Cape Town, South Africa; ^2^TB Vaccine Group, Jenner Institute, University of Oxford, Oxford, UK; ^3^Lung Infections and Immunity Unit and UCT Lung Institute, Division of Pulmonology, Department of Medicine, Groote Schuur Hospital and University of Cape Town, Cape Town, South Africa; ^4^Institute of Infectious Diseases and Molecular Medicine, University of Cape Town, Cape Town, South Africa

## Methods

### Diagnostic classification for analysis

All included patients had a diagnosis of a pericardial syndrome, with echocardiographic confirmation of a pericardial effusion. Patients were categorised into the following diagnostic groups:

1. Definite-TB: *M. tb* sample positive by liquid culture (either pericardial or non-pericardial) and/or granulomatous inflammation on pericardial tissue histology (i.e., composite reference standard).
2. Non-TB: No microbiological evidence of *M. tb* and an alternative diagnosis is made.
3. Probable-TB: Clinical and / or biochemical diagnosis characterised by elevated ADA and lymphocytic predominance; not meeting the criteria for definite-TB.

### LAM strip test methodology

All patients were required to give two spot urine samples (50 to 100ml) collected in a sterile container at enrollment. Urine was stored at -20°C for later batched testing. The LAM strip test was performed on unprocessed urine according to manufacturer’s instructions. Briefly, urine was thawed, mixed and 60μl pipetted onto the lateral flow strip loading bay. After 25 minutes, two readers in ambient laboratory lighting conditions, and blinded to the clinical patient details and clinical TB status, independently evaluated the LAM strips for all study patients via the following procedure: after confirming test validity by identifying the presence of a band in the positive control window, the intensity of the colour band (if any) in the patient window was read by comparison with the manufacturer-provided visual reference scale card (graded 0 – 5 depending on band intensity). Using the manufacturer-recommended grade 1 cut-point, a band of visual intensity ≥ grade 1 in the patient window was classified test ‘positive’ while only the complete absence of a band (grade 0) in the patient window was classified test ‘negative’. Accuracy was assessed at various alternative cut-points to select one for optimal rule-in value. For example, if the grade 2 cut-point was selected the complete absence of band (grade 0) as well as a faint band (grade 1) was classified test ‘negative’ and only a band of visual intensity ≥ grade 2 was considered test ‘positive’. The test was reported as indeterminate if a broken/ incomplete band was seen in the patient window. A test was reported as failed if no control band was identified. Each reader graded the LAM strips blinded to the results of the other. The results were then compared and if discrepant, a third reader was asked to independently grade the strip and the consensus result was used.

### **Figure S1**: Pre-January 2014 LAM strip test manufacturer’s reference card illustrating visual intensity grades 0-5

### Reproduced with permission of the European Respiratory Society ©: European Respiratory Journal Nov 2012, 40 (5): 1211-1220; DOI: 10.1183/09031936.00201711. Permission granted by Alere to publish this figure.

### **Figure S2**: Screening, recruitment and diagnostic classification

###

### Supplementary Table S1: Diagnostic accuracy measures for urinary and pericardial fluid LAM (measured by ELISA and strip test). Definite-TB and Probable-TB used for sensitivity and Non-TB used for specificity calculations

| **+**  **Diagnostic Test** | **Patient group** | **Sensitivity**  **(95% CI)**  **(n/N)** | **Specificity**  **(95% CI)**  **(n/N)** | **Positive predictive value, PPV (95% CI)** | **Negative predictive value, NPV (95%CI)** |
| --- | --- | --- | --- | --- | --- |
| **Urine LAM ELISA** | **All patients** | 14.6%  (8.6-23.9)  12/82 | 100%  (78.5-100)  14/14 | 100%  (75.8-100) | 16.7% (10.2-26.1) |
|  | **HIV positive** | 17.9%  (10.6-28.8)  12/67 | 100%  (34.2-100)  2/2 | 100%  (75.8-100) | 3.5%  (1.0-11.9) |
|  | **CD4 ≤100 cells/mm^3^** | 45.5%  (26.9-65.3)  10/22 | Undefined  0/0 | 100%  (72.3-100) | 0.0%  (0.0-24.3) |
|  | **CD4 >100 cells/mm^3^** | 2.4%  (0.4-12.6)  1/41 | 100%  (34.2-100)  2/2 | 100%  (20.7-100) | 4.8%  (1.3-15.8) |
| **Urine LAM strip test (grade 2 cut-point)** | **All patients** | 18.5%  (11.6-28.3)  15/81 | 92.9%  (68.5-98.7)  13/14 | 93.8%  (71.7-98.9) | 16.5%  (9.9-26.2) |
|  | **HIV positive** | 21.2%  (13.1-32.5)  14/66 | 100%  (34.4-100)  2/2 | 100%  (78.5-100) | 3.7%  (1.0-12.5) |
|  | **CD4 ≤100 cells/mm^3^** | 45.5%  (26.9-65.3)  10/22 | Undefined  0/0 | 100%  (72.3-100) | 0.0%  (0.0-24.3) |
|  | **CD4 >100 cells/mm^3^** | 7.5% (2.6-19.9)  3/40 | 100%  (34.4-100)  2/2 | 100%  (43.9-100) | 5.1%  (1.4-16.9) |
| **PF LAM ELISA** | **All patients** | 10.5%  (6.1-17.5)  12/114 | 88.5%  (71.0-96.0)  23/26 | 80.0%  (54.8-93.0) | 18.4%  (12.6-26.1) |
|  | **HIV positive** | 10.9%  (6.0-18.9)  10/92 | 100%  (51.0-100)  4/4 | 100%  (72.3-100) | 4.7%  (1.8-11.4) |
|  | **CD4 ≤100 cells/mm^3^** | 21.2%  (10/7-37.8)  7/33 | Undefined  (0/0) | 100%  (64.6-100) | 0.0%  (0.0-12.9) |
|  | **CD4 >100 cells/mm^3^** | 5.5%  (1.9-164.9)  3/55 | 100%  (51.0-100)  4/4 | 100%  (43.9-100) | 7.1%  (2.8-17.0) |
| **PF LAM strip test (grade 2 cut-point)** | **All patients** | 15.3%  (9.8-23.2)  17/111 | 88.5%  (71.0-96.0)  23/26 | 85.0%  (64.0-94.8) | 19.7%  (13.5-27.8) |
|  | **HIV positive** | 14.4%  (8.6-23.2)  13/90 | 100%  (51.0-100)  4/4 | 100%  (77.2-100) | 4.9%  (1.9-12.0) |
|  | **CD4 ≤100 cells/mm^3^** | 27.3%*§  (15.1-44.2)  9/33 | Undefined  0/0 | 100%  (70.1-100) | 0% (0.0-13.8) |
|  | **CD4 >100 cells/mm^3^** | 7.6%*§  (3.0-17.9)  4/53 | 100%  (51.0-100)  4/4 | 100%  (51.0-100) | 7.5%  (3.0-17.9) |
| **PF uIFNγ (Intergam) (Youden’s, rule-in and rule-out cut-points: ≥44pg/ml)** | **All patients** | 92.1%* (85.7-95.8) 105/114 | 96.3% (81.7-99.3) 26/27 | 99.1% (94.9-99.8) | 74.3% (57.9-85.8) |
|  | **HIV positive** | 91.3%* (83.8-95.5) 84/92 | 80% (37.6-96.4) 4/5 | 98.8%  (93.6-99.8) | 33.3%  (13.8-60.9) |
| **PF ADA (Cut-point in current clinical use: ≥35 IU/ml)** | **All patients** | 84.6%* (77.0-90.0) 99/117 | 84% (65.4-93.6) 21/25 | 96.1% (90.4-98.5 | 53.9% (38.6-68.4) |
|  | **HIV positive** | 83.9%* (75.1-90.0) 78/93 | 40%¶ (11.8-76.9) 2/5 | 96.3%  (89.7-98.7) | 11.8%  (3.3-34.3) |

## Acknowledgements

We thank the research, nursing, and clinical staff of the following institutions for the roles they played in patient referral, recruitment and management: Groote Schuur Hospital Cardiac Clinic, Cardiac Catheterisation Laboratory, and C15 Emergency Unit; University of Cape Town Lung Infection and Immunity Research Unit; GF Jooste, New Somerset, and Victoria Hospitals. We acknowledge the following multiple funding sources that have contributed to the project: (i) Discovery Foundation Fellowships (to Drs S. Pandie and J Peter); (ii) Medical Research Council of South Africa; (iii) The Lily and Ernst Hausmann Research Trust; (iv) South African National Research Foundation; (v) A TBSusgent grant from the European Commission (EU-FP7); (vi) the National Institutes of Health grant (R24TW007988); and (vii) the European and Developing Countries Clinical Trials Partnership (EDCTP; TB-NEAT and TESA).
